# Alterations in Salience Network Functional Connectivity in Individuals with Restless Legs Syndrome

**DOI:** 10.1038/s41598-020-64641-w

**Published:** 2020-05-06

**Authors:** Jeonghun Ku, Yeong Seon Lee, Keun Tae Kim, HyukWon Chang, Yong Won Cho

**Affiliations:** 1 Department of Biomedical Engineering, Keimyung University School of Medicine, Dongsan Medical Center, Daegu, South Korea; 2 Department of Neurology, Keimyung University School of Medicine, Dongsan Medical Center, Daegu, South Korea; 3 Department of Radiology, Keimyung University School of Medicine, Dongsan Medical Center, Daegu, South Korea

**Keywords:** Sleep disorders, Magnetic resonance imaging

## Abstract

Background and purpose: Restless legs syndrome (RLS) is a neurological disorder which is most commonly identified by an urge to move the legs. It often shows alterations in sensory processing which implies the salience network (SN) is experiencing changes. This study investigates the functional connectivity (FC) between the SN and other areas of the brain in RLS patients during the resting state period. Methods: Thirty patients with drug naïve idiopathic RLS and 30 healthy age and gender matched controls were included in this study. Resting state fMRIs were performed in the morning during the asymptomatic period. The SN comparisons were conducted between the two groups. Results: The RLS group showed a reduction in SN FC in the right pyramis, and an increase in SN FC in the bilateral orbitofrontal gyri and right postcentral gyrus. Conclusions: The results of this study give reason to believe that SN FC in RLS patients is altered during asymptomatic periods. This could have an influence on the processing of the saliency of information, particularly sensory information processing and inhibition mechanisms.

## Introduction

Restless legs syndrome (RLS) is a neurological disorder in which patients often report an urge to move the legs (i.e., akathisia)^1^. The prevalence of RLS is 3.9–11.5% in the general population^2,3^. Two major putative causes for RLS are dopaminergic abnormality and iron insufficiency in the brain. Pharmacological therapy commonly includes drugs, such as dopamine agonists, iron, benzodiazepines, opiates, and alpha-2-delta ligands^4^. Leg akathisia is typically triggered by rest or a reduction in arousal^1^. It leads to alterations in the sensory processing mechanism of peripheral and brain responses. Brain imaging studies showed that there are morphologic changes in the primary somatosensory system^5^. Additionally, fMRI studies conducted on the thalamic and default mode networks have shown clear differences when compared to healthy subjects^6,7^, with varying results throughout the day^8^. In addition, other functional connectivity studies suggest that RLS is associated with impaired attentional control of sensory input^9^. A neurophysiological study showed that RLS patients have a deficit of inhibitory modulation of the primary motor cortex^10^. Those studies consistently suggest that RLS can alter sensory information processing and sensory motor integration, and can activate the brain’s compensation mechanism to inhibit sensations. These alterations may lead to abnormal sensory information processing, so RLS patients may experience unusual sensations and show lower current perception threshold (CPT) values. The studies also suggest impaired central sensory processing in RLS patients^11^. In addition, electrophysiological evidence suggests that sensory processing deficits are particularly related to the excitability of the early somatosensory gating control and an attenuated inhibitory interneuron network^12^. Thus, RLS patients may be more sensitive in perceiving sensations than the average person.

All these sensory processing deficits can be influenced by alterations in the patient’s perception of saliency. In RLS patients, non-salient events seem to be coded in the brain as salient, so the patient cannot properly identify salient stimuli as opposed to non-salient stimuli^13^. This may result in the subject having a lower threshold for perceiving sensory stimuli^11^.

The Salience Network (SN) is the part of the brain that detects and filters salient stimuli and recruits relevant functional networks^14^. It plays a role in detecting and integrating emotional and sensory stimuli, while simultaneously switching between the internally directed cognition of the default mode network and the externally directed cognition of the central executive network (CEN).

Using this perspective of the role of the SN, it could be assumed that the SN influences the other brain networks including the processing of sensory information in RLS patients. Perceptions traveling through the SN may possibly be altered and the connectivity of brain regions with the SN may also be altered, which would allow RLS symptoms to develop as the network attempts to process excessively non-salient stimuli. The purpose of this study was to investigate the alterations of the SN FC in RLS patients during the resting state to obtain further insight into the pathophysiology of RLS.

## Methods

### Study population

This study recruited 30 idiopathic RLS patients who had never received drug treatment for their condition (i.e, drug naïve) who visited a tertiary hospital outpatient sleep disorder center. It also included 30 age and gender matched healthy control subjects who had not experienced any previous sleep problems or other medical disorders. The control subjects all answered “no” on the initial questions of the RLS Diagnostic Questionnaire^3,15^. All patients were individually diagnosed by a certified Korean neurologist and RLS expert (YWC) through face-to-face interviews which utilized the Korean-language version^3^ of the Johns Hopkins Telephone diagnostic questionnaire^15^. All mimic diseases were excluded, as recommended by the updated international restless legs syndrome study group (IRLSSG) diagnostic criteria^16^. Patients diagnosed with secondary RLS because of iron-deficiency anemia, pregnancy, chronic kidney disease, peripheral neuropathy, or myelopathy were excluded. However, there were some cases of patients who only had peripheral iron deficiency with no definite cause. These patients were included in the study and their condition was not controlled for as with other RLS studies. All patients were untreated prior to enrolling in this study. The severity of RLS symptoms were evaluated using the validated Korean-language version^17^ of the International RLS scale (K-IRLS)^18^. Patients with circadian sleep disorders, parasomnia, sleep apnea, or other comorbid sleep disorders were identified using validated Korean-language versions of sleep questionnaires, including the Insomnia Severity Index (ISI-K)^19^, the Pittsburgh Sleep Quality Index (PSQI-K)^20^, and the Epworth Sleepiness Scale (ESS-K)^21^. Polysomnography was performed if needed, and all patients who reported comorbid sleep disorders were excluded.

The study was approved by the institutional review board of the Keimyung University Dongsan Hospital. Informed consent was obtained from all participating subjects. All methods were carried out in accordance with relevant guidelines and regulations.

### MRI protocol

Resting state fMRI scans were conducted in the morning between 09:00–12:00, in order to minimize influence from RLS symptoms. A 3 T MRI Signa Excite scanner (GE Healthcare, Milwaukee, WI, USA) with an 8-channel high-resolution brain coil was used for this study. Each subject underwent an anatomic image series scan using a three-dimensional spoiled gradient-echo sequence (repetition time (TR) = 6 ms, echo time (TE) = 2.2 ms, flip angle = 20°, field of view = 240 mm, 256 ×256, 152 axial slices, slice thickness = 2 mm thick). The functional images were scanned using a gradient echo planar imaging (EPI) sequence (TR = 2000 ms, TE = 17.6 ms, flip angle = 90°, field of view = 240 mm, matrix = 64 ×64, slice thickness = 4 mm, no gap, 244 scans of 30 contiguous axial slices) for approximately 8 minutes. Subjects were instructed to close their eyes without sleeping while maintaining a resting state for the duration of the scan. Patients who were unable to do so would be excluded, although there were no such instances during this study. In addition, none of the patients in this study reported RLS symptoms immediately before, during, or immediately after the MRI was administered.

### Analysis of resting-state fMRI

Resting-state fMRI data was analyzed using the Analysis of Functional Neuroimage (AFNI) software^22^. The time series dataset discarded the first four time points, as is standard. The analysis included slice time correction for interleaved acquisitions, despiking, three-dimensional motion correction (allowing for head movement of <2.5 mm), temporal normalizing, linear and quadratic detrending, spatial normalization using the Montreal Neurological Institute (MNI) 152 template provided in the AFNI package, spatial smoothing (full width at half maximum 6 mm), and temporal filtering (0.009–0.1 Hz).

**Figure 1:**
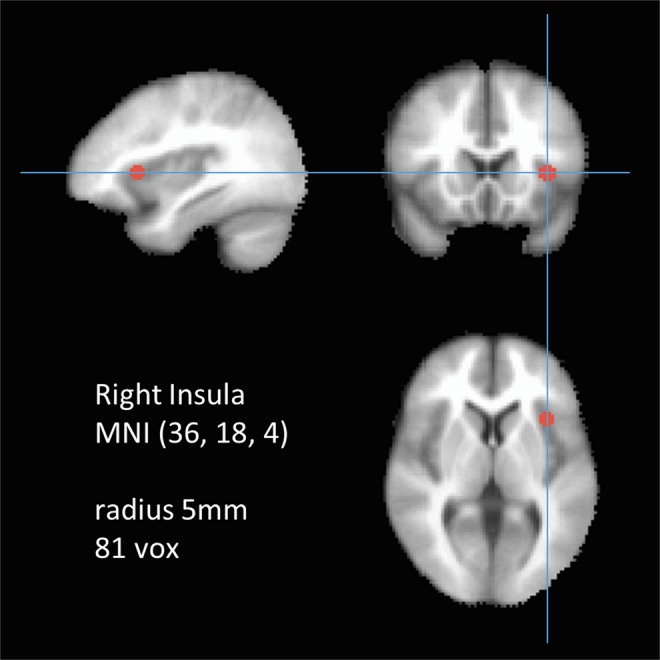
The seed region of interest was marked as 5 mm radius in the right insula at (36, 18, 4) in MNI coordinates.

The target region was defined as a 5 mm seed sphere in the right anterior insula (Fig. 1) because the right anterior insula has been previously identified as a crucial node of the SN^23^.Because there is no standard seed size, and typical variation is between 4 mm^24^ and 6 mm^25^, the seed was set as 5 mm. Whole-brain voxel-wise correlations associated with the mean time series for the right insula were derived for each participant using 3dDeconvolve (AFNI). For this step, the software identified and regressed 27 separate predictor modeling nuisance signals (white matter, cerebrospinal fluid, global signal, and 24 motion parameters^26–28^). Using the motion parameters, framewise displacement (FD)^29^ was calculated and the average value of the FD was used as covariates in the group analysis step. The FD values were between 0.0412 and 0.2570 so nobody was excluded due to head movement. Each participant’s correlation maps were converted to z score maps (Fisher r-to-z transformation) representing the strength of FC between brain areas and the seed region, which were then used to analyze group data.

An analysis of covariance (adjusted for FD, HAS and HDS) was used to evaluate differences in SN FC in the control group as compared to RLS patients. The 3dClustSim command provided in the AFNI analysis program was used to get the corrected p value of 0.05 with the threshold value of the cluster size being 800 microliters (100 voxels) under the uncorrected p value of 0.01. The strength of the connectivity from each brain region was compared with the patients’ characteristics, which included symptom duration, age of onset, K-IRLS total, and sub-scores of K-IRLS^30^ (significance for these exploratory analyses was set at *p* < 0.05).

## Results

The mean age of the RLS patients and controls was 53.8 ± 10.6 and 53.4 ± 11.2, respectively. There were 23 RLS females (76.7%) and 19 control females (63.3%). The RLS and control groups did not differ significantly in age or gender (Table 1). The mean (±sd) K-IRLS was 26.4 ± 6.9, and the mean (±sd) RLS duration was 133.4 ± 134.9 months. A summary of the sleep assessments for the RLS control groups is included in Table 1 (Figs. S1–9). RLS patients and controls showed no abnormalities, nor any brain atrophy on their T1, T2, or diffusion images, as confirmed by a neuro-radiologist.

**Table 1:** Demographic and clinical characteristics.

	Drug naïve RLS patients (n = 30)	Controls (n = 30)	χ^2^/*t*	*p*-value
Females (%) / males (%)	23(76.7) / 7(23.3)	19(63.3) / 11(36.7)	1.270	0.260
Age (years)	53.8 ± 10.6	53.4 ± 11.2	0.142	0.887
RLS severity (K-IRLS)	26.4 ± 6.9			
symptom severity	19.3 ± 4.8			
disease-specific QoL	9.3 ± 3.1			
Age of onset	43.2 ± 15.0			
Symptom duration (month)	133.4 ± 134.9			
PSQI-K	11.1 ± 4.0	4.0 ± 1.4	9.143	<0.001
ISI-K	16.2 ± 6.7	3.5 ± 2.5	9.792	<0.001
ESS-K	6.3 ± 4.6	4.5 ± 3.3	1.51	0.138
HAS	7.7 ± 4.3	3.5 ± 1.9	4.919	<0.001
HDS	8.3 ± 3.7	5.1 ± 2.7	3.863	<0.001

The values are mean ± SD.

RLS: restless legs syndrome, K-IRLS: Korean version of the international RLS severity scale, QoL: quality of life, PSQI-K: Korean version of the Pittsburgh sleep quality index, ISI-K: Korean version of insomnia severity index, ESS-K: Korean version of Epworth sleepiness scale, HAS: Hospital Anxiety Scale, HDS: Hospital Depression Scale.

**Figure 2:**
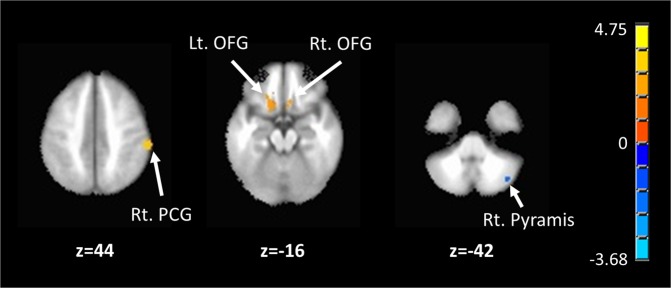
The areas showed differences the connectivity of the right anterior insula between the RLS group and healthy controls. The blue color represents the reduced connectivity of the SN, while the red areas indicate increased connectivity of the SN in the RLS patients. Lt., left; Rt., right; OFG, orbitofrontal gyrus; PCG, postcentral gyrus.

During the asymptomatic period, the RLS group showed a reduction in SN connectivity in the right pyramis (Group mean and std. values across subjects of FC values across all voxels in the cluster were −0.1617 ± 0.1232 for RLS and −0.0159 ± 0.1466 for controls, which is placed at (40, −68, −42) in the MNI coordinate with 1040 cluster size showing Max t value of −3.5817). There was an increase in SN connectivity for the bilateral orbitofrontal gyri (Group mean and std. values across subjects of FC values across all voxels in the cluster were 0.1527 ± 0.1174 for RLS and 0.0140 ± 0.0988 for controls, which is placed at (−14, 24, −18) in the MNI coordinate with 2232 cluster size showing Max t value of 3.8768 for the left; Group mean and std. values across subjects of FC values across all voxels in the cluster were 0.1197 ± 0.1544 for RLS and −0.0403 ± 0.1855 for controls at (6, 18, −14) in the MNI coordinate with 856 cluster size showing Max t value of 3.6056 for the right) and right postcentral gyrus compared to healthy controls(Group mean and std. values across subjects of FC values across all voxels in the cluster were 0.3077 ± 0.2083 for RLS and 0.1176 ± 0.2073 for controls at (62, −32, 44) in MNI coordinate with 1400 cluster size showing Max t value of3.7687) (Table 2, Fig. 2).

**Table 2:** Patterns of connectivity in restless legs syndrome.

**Anatomical region**	**#Volume**	**X**	**Y**	**Z**	**Max** ***t***
**lower connectivity in RLS**					
Right Pyramis	1040	40	−68	−42	−3.5817
**higher connectivity in RLS**					
Left Orbitofrontal Gyrus	2232	−14	24	−18	3.8768
Right Postcentral Gyrus	1400	62	−32	44	3.7687
Right Orbitofrontal Gyrus	856	6	18	−14	3.6056

**Figure 3:**
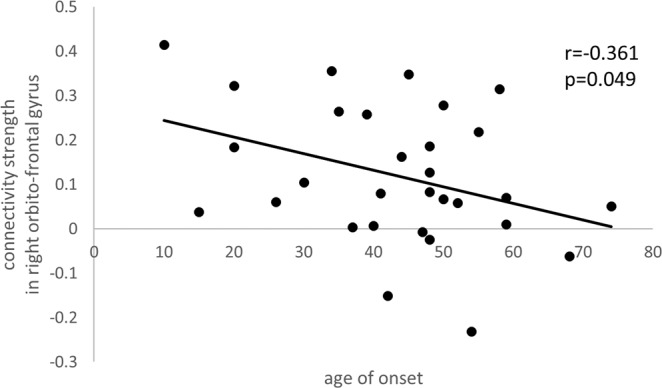
The correlation between the connectivity strength in the right orbitofrontal gyrus and age of onset in RLS patients. The x-axis is age of onset and the y-axis is connectivity strength in the right orbito-frontal gyrus.

**Figure 4:**
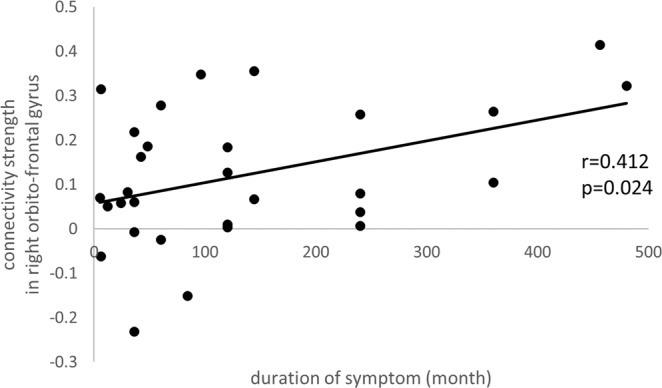
The correlation between the connectivity strength in the right orbitofrontal gyrus and duration of symptoms in RLS patients. The x-axis is the duration of symptoms and the y-axis is connectivity strength in the right orbito-frontal gyrus.

There were two significant correlations when analysis was conducted between the patients’ characteristics and the connectivity strength of the right orbitofrontal gyrus. The age of onset was negatively correlated with connectivity strength between the right anterior insula and right orbitofrontal gyrus (r = −0.361, p = 0.049) (Fig. 3) and the duration of symptoms was positively correlated with connectivity strength between the right anterior insula and right orbitofrontal gyrus (r = 0.412, p = 0.024) (Fig. 4). Connectivity strength was not correlated with any other characteristics such as K-IRLS total (r = −0.007, p = 0.971), or sub-scores of K-IRLS (r = 0.001, p = 0.997). However, the significant correlations were not maintained when multiple comparisons were considered.

## Discussion

This study showed several changes in SN FC, particularly in the right anterior insula of RLS patients. The research team operated under the hypothesis that the SN plays a determining factor in identifying salient stimuli, which in turn could influence RLS symptoms. One suggestion is that the identification of salient stimuli may be incorrectly processed in RLS patients, resulting in alterations in SN FC. Test results showed increased SN FC in the right postcentral gyrus and bilateral orbitofrontal gyrus, while SN FC in the cerebellum decreased.

### Functional connectivity with the salience network

The SN usually functions as a switch between the default mode network and the executive network by detecting salient events and coordinating other brain networks^14,33,34^. According to Putch’s review^34^, the SN and the Central Executive Network (CEN) increase in activity when saliency is detected and cognitive tasks which require attention to external stimuli are present^35^, whereas DMN activity is suppressed^36,37^. The SN is thought to be responsible for detecting and filtering information necessary to maintain goal-directed behavior by shifting attention between external and internal processing^38,39^. Therefore, the alteration of FC with the SN could lead to the failure of the switching or filtering processes, so the sensory related salience signals which are generated internally would be detected by the SN and improperly processed by other brain networks (e.g. executive function). Therefore, the fact that the brain areas in this study showed aberrant connectivity with the right anterior insula compared to healthy controls could be viewed as a result of incorrect processing of saliency, or as a change in FC with the SN. If the anterior insula is unable to handle this role on its own, it could lead to the recruitment of other brain networks to process the information which might also result in flawed or incomplete processing after being detected by the SN.

The postcentral gyrus is a representative area for somatosensory processing and receives the bulk of the thalamocortical projections from the brain’s sensory input fields^31^. So, the enhanced connectivity between the right anterior insula (associated with awareness of viscerosensory information^32^ and salience processing in inhibitory control^33^) and the postcentral gyrus could mean that salience stimuli are closely connected to the somatosensory area in individuals with RLS. This area can process sensory information when RLS patients perceive and inhibit sensory stimuli. One possible explanation is that when sensory-related saliency is generated internally, it can be easily detected and transferred to the somatosensory areas, which would then resolve and inhibit said saliency.

The orbitofrontal area is one of the representative areas for the executive function and is usually involved in inhibition^33,40^. It plays a key role in error monitoring and correction. Damage to the orbitofrontal cortex in humans can hinder learning and reverse stimulus-reinforcement associations. In addition, damage to this area has been shown to result in reduced control over emotions, limited awareness of the moral implications of actions, and poor decision making^41,42^. The orbitofrontal cortex is also activated to resolve ambiguity. Internally generated stimuli, which could include the urge to move in RLS patients, are likely to increase ambiguity in processing. Therefore, the activation of the orbitofrontal network would be involved in order to resolve this ambiguity. It also represents secondary salience signals that reflect body sensations by way of the medial orbitofrontal network (or ‘visceromotor’) which helps control internal body states^43^.

Increased FC with the SN is regularly observed in the sleep-deprived brain^44^ as well as patients with insomnia who are in hyper-arousal state^45^. Therefore, it is possible that the alterations in SN FC as witnessed by our team may be associated with the hyper-arousal state that can occur as a result of RLS^46^^.^

### Limitations

Although there are valuable findings obtained in this study, there are several limitations as well. First, research was conducted with a small sample size, so a firm conclusion is difficult to reach at this time. Additionally, only the RLS group was compared to the controls even though there may be other patient groups which share similar brain mechanism dysfunctions (e.g. somato-sensory or sleep disorders) as those with RLS. Sleep quality and mood swings in RLS patients may also affect the results. Finally, our study only conducted resting-state functional connectivity analysis using fMRI. The limitations listed above mean that results of this study may be vulnerable to misinterpretation or generalization because of their lack of specificity. Additional multi-modal approaches would help to provide clarification and alternate perspectives. Therefore, further research would be necessary to resolve these issues and provide a comprehensive outline of the topic at hand.

## Conclusion

Despite these limitations, the findings of this study suggest that the FC with the SN in RLS patients may be associated with several changes in the overall FC of the brain, which could have an influence on the ability to process the saliency of information. We confirmed that other brain areas were activated together for the additional processing of information. This is particularly relevant when considering sensory information processing and inhibition mechanisms. It could give valuable insight for understanding the pathophysiology of RLS patients.

## Supplementary information

Supplementary Information.
