# Supplementary material for: Alterations in Salience Network Functional Connectivity in Individuals with Restless Legs Syndrome
Source: Sci Rep. 2020 May 6;10:7643. doi: 10.1038/s41598-020-64641-w (PMC7203171; doi:10.1038/s41598-020-64641-w)
Supplement: Supplementary file 1 — Supplementary Information. [file 41598_2020_64641_MOESM1_ESM.docx]

**Supplementary Information**

Alterations in Salience Network Functional Connectivity in Individuals with Restless Legs Syndrome

Jeonghun Ku, PhD^1^, Yeong Seon Lee^2^, Keun Tae Kim, M.D.^2^,

HyukWon Chang, M.D.^3^, Yong Won Cho, M.D.^2^*

Department of ^1^Biomedical Engineering, ^2^Neurology, and ^3^Radiology, Keimyung University School of Medicine, Dongsan Medical Center, Daegu; South Korea;

*author to whom correspondence should be addressed: neurocho@gmail.com

**Content**

1. Histograms for demographic and clinical characteristics in the restless legs syndrome patients and controls

Figure S1: Age distribution of the RLS patients and controls

Figure S2: Distribution of symptom severity in the RLS patients

Figure S3: Distribution of age of onset in the RLS patients

Figure S4: Distribution of symptom duration in the RLS patients

Figure S5: Distribution of the Korean version of the Pittsburgh sleep quality index scores in the RLS patients and controls

Figure S6: Distribution of the Korean version of the insomnia severity index scores in the RLS patients and controls

Figure S7: Distribution of the Korean version of the Epworth sleepiness scale scores in the RLS patients and controls

Figure S8: Distribution of the hospital anxiety scale scores in the RLS patients and controls

Figure S9: Distribution of the hospital depression scale scores in the RLS patients and controls

1. Histograms for demographic and clinical characteristics in the restless legs syndrome patients and controls


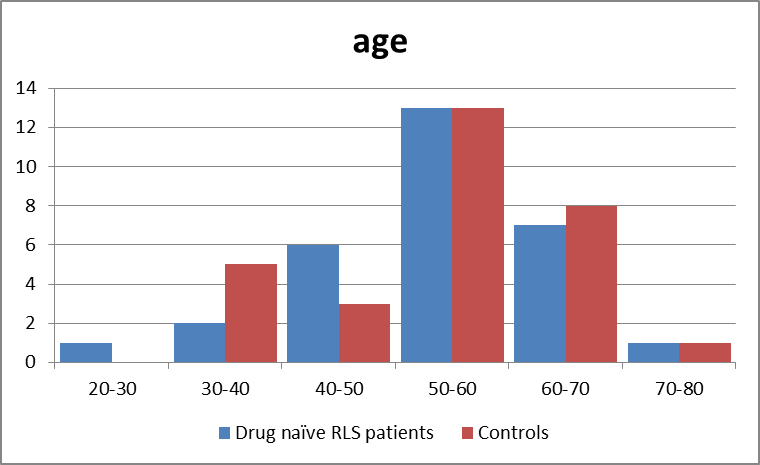


Figure S1: Age distribution of the RLS patients and controls

Figure S2: Distribution of symptom severity in the RLS patients


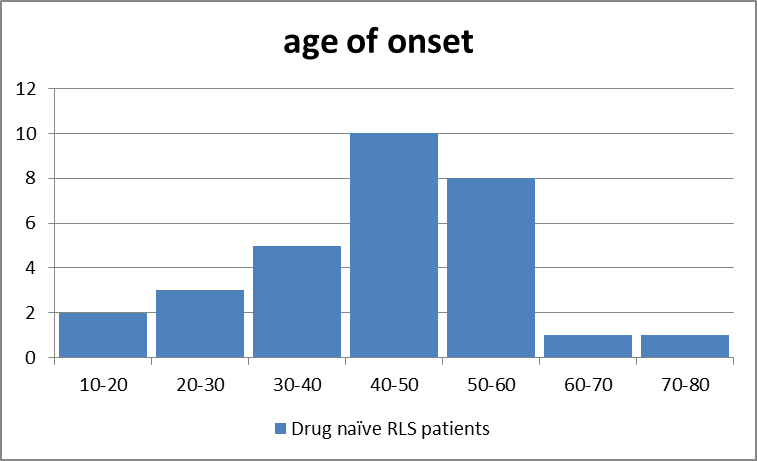


Figure S3: Distribution of age of onset in the RLS patients

Figure S4: Distribution of symptom duration in the RLS patients


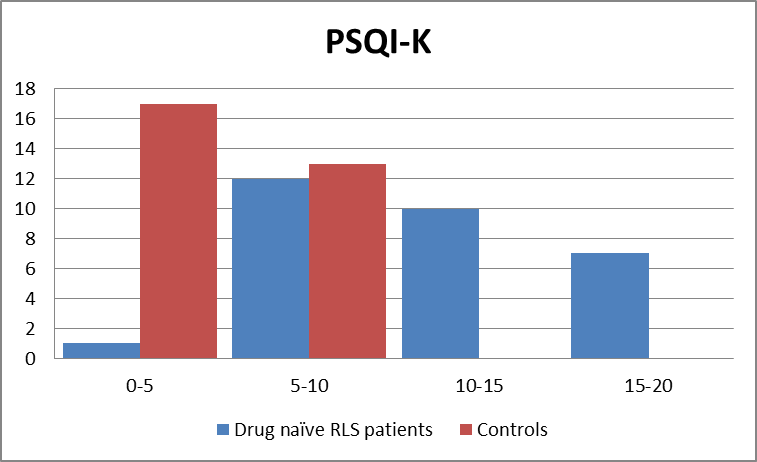


Figure S5: Distribution of the Korean version of the Pittsburgh sleep quality index scores in the RLS patients and controls


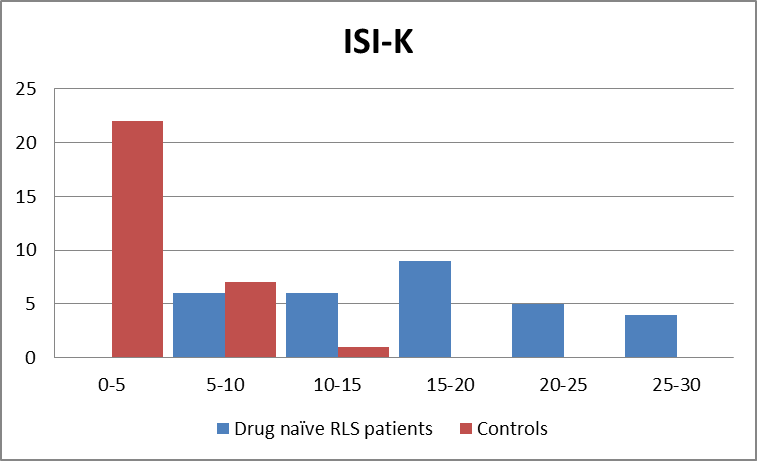


Figure S6: Distribution of the Korean version of the insomnia severity index scores in the RLS patients and controls


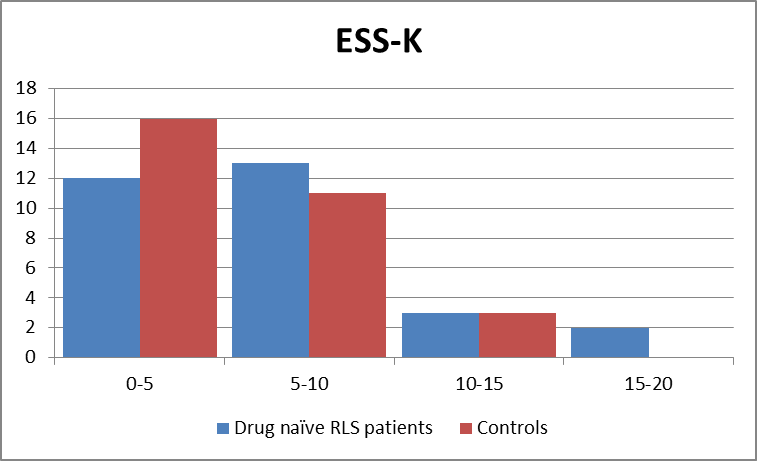


Figure S7: Distribution of the Korean version of the Epworth sleepiness scale scores in the RLS patients and controls


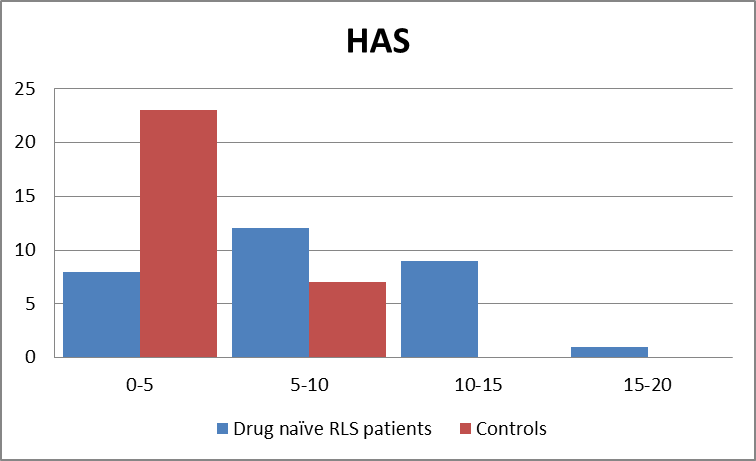


Figure S8: Distribution of the hospital anxiety scale scores in the RLS patients and controls


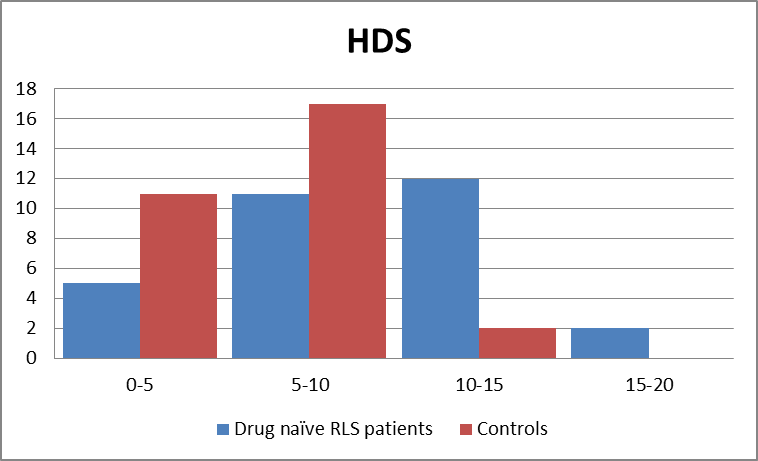


Figure S9: Distribution of the hospital depression scale scores in the RLS patients and controls
